# Evidence for the formation of two types of oxygen interstitials in neutron-irradiated α-Al_2_O_3_ single crystals

**DOI:** 10.1038/s41598-021-00336-0

**Published:** 2021-10-22

**Authors:** A. Lushchik, V. N. Kuzovkov, E. A. Kotomin, G. Prieditis, V. Seeman, E. Shablonin, E. Vasil’chenko, A. I. Popov

**Affiliations:** 1 Institute of Physics, University of Tartu, W. Ostwald Str. 1, 50411 Tartu, Estonia; 2 Institute of Solid State Physics, University of Latvia, Kengaraga 8, Riga, 1063 Latvia

**Keywords:** Materials science, Physics

## Abstract

Due to unique optical/mechanical properties and significant resistance to harsh radiation environments, corundum (α-Al_2_O_3_) is considered as a promising candidate material for windows and diagnostics in forthcoming fusion reactors. However, its properties are affected by radiation-induced (predominantly, by fast neutrons) structural defects. In this paper, we analyze thermal stability and recombination kinetics of primary Frenkel defects in anion sublattice − the *F*-type electronic centers and complementary oxygen interstitials in fast-neutron-irradiated corundum single crystals. Combining precisely measured thermal annealing kinetics for four types of primary radiation defects (neutral and charged Frenkel pairs) and the advanced model of chemical reactions, we have demonstrated for the first time a co-existence of the two types of interstitial defects – neutral O atoms and negatively charged O^-^ ions (with attributed optical absorption bands peaked at energies of 6.5 eV and 5.6 eV, respectively). From detailed analysis of interrelated kinetics of four oxygen-related defects, we extracted their diffusion parameters (interstitials serve as mobile recombination partners) required for the future prediction of secondary defect-induced reactions and, eventually, material radiation tolerance.

## Introduction

Aluminium oxide, α-Al_2_O_3_ (corundum, space group $R\overline{3 } c$), reveals high melting temperature, wide optical transparency region and other exceptional properties. It finds numerous applications in microelectronics and aerospace industry, as laser media and materials for optics, luminescent dosimetry and cryogenic scintillators^1–4^. Because of the ability to maintain mechanical and electric integrity, rather low swelling and high resistance against fast neutron irradiation, α-Al_2_O_3_ single crystals and polycrystalline ceramics are widely used in fission-based nuclear energetics (coatings, moderators, absorbers, waste disposal)^5,6^ and are in the short list of promising window materials for projected fusion devices^5–7^.

It is obvious that functionality of many materials is limited by insufficient tolerance to heavy intentional radiation or even prolonged stay in harsh environment. By the modern concepts, radiation resistance of the material mainly depends on the accumulation of stable (long-lived, *τ* >  > 1 s) lattice defects, primary Frenkel pairs (interstitial-vacancy) and their aggregates. Note that the efficiency of radiation damage in wide-gap metal oxides, which are characterized by rather high formation energy of the Frenkel defect pair (noticeably exceeds the energy gap, *E*_FD_ > *E*_g_), strongly depends on radiation type. For instance, conventional X- and γ-rays do not create new structural defects in metal oxides, but only provide the recharging of already existing as-grown defects and impurities by the induced charge carries (such radiation creates mainly rapidly recombining electron–hole pairs).

On the other hand, elastic collisions of incident energetic, above-threshold particles (fast neutrons, electrons, ions) with material nuclei determine the formation of radiation damage in oxides^8–12^. Similar to metals and alloys, such displacement (collision, impact) mechanism solely describes the creation of Frenkel defects by fast neutrons in metal oxides^8,9,12–14^. At the same time, a significant part of swift heavy ion energy is spent on ionization losses, i.e. the formation of very high density of electronic excitations along ion path (> 20 keV/nm for ~ GeV-ions)^15,16^. In the majority of alkali halides, *E*_FD_ < *E*_g_ and Frenkel defects are efficiently formed via the so-called ionization mechanisms: the nonradiative decay of self-trapped anion excitons (their formation energy is even lower than *E*_g_) or recombination of totally relaxed electron–hole pairs that provides the released energy around *E*_g_ (see ^17^). Because of opposite energetic inequality, *E*_FD_ > *E*_g_ in metal oxides, only additional electronic-excitation-related mechanisms, more complex than those in model alkali halides^17^, to some extent, contribute to radiation damage of oxides under swift heavy ion irradiation (see ^16,18–21^ and references therein).

Experimental studies of radiation processes caused by α-Al_2_O_3_ exposure to radiation of different types − fast fission neutrons, energetic electrons or heavy ions—have been performed for many years^21–36^. The optical characteristics (optical absorption and emission bands) of the main point lattice defects—the so-called *F*^+^ and *F* centers, which represent an oxygen vacancy with one or two captured electrons, respectively, have been thoroughly studied in irradiated and additively colored (thermochemically reduced) corundum crystals^24–28,37^. Because of its paramagnetic nature, the *F*^+^ center has also been studied by the electron paramagnetic resonance (EPR) method in the α-Al_2_O_3_ crystal irradiated with fast neutrons as well^23^. In addition, the simplest aggregates of *F*-type centers, two adjacent oxygen vacancies - *F*_2_ dimers in different charge states have been also detected and studied in corundum^28–30,38^.

On the other hand, an interstitial oxygen (neither in a neutral nor charged state), which constitutes the complementary to an anion vacancy Frenkel defect, for a long time remained the most hidden primary point defect in wide-gap metal oxides (see ^26,39–41^). Only recently, the EPR signal ascribed to the oxygen interstitial in a form of a superoxide ion O_2_^−^ without any other defect/impurity in its vicinity has been revealed in a fast-neutron-irradiated α-Al_2_O_3_ single crystal^42^. This experimental finding was in a good agreement with the first principles calculations of the atomic, electronic and magnetic structure of the defect. Note that first principles calculations of both interstitial oxygen atoms^43,44^ and ions^45^ in corundum predict that in the ground state they form the O_2_ molecular ions (*dumbbells*) with different charges, their diffusion occurs via bond breaking with formation of the *O* atom or *O*^-^ ions at the saddle point of a jump. The estimated migration energies were around 1.2 eV and 0.8 eV, respectively. The oxygen dumbbells are similar to the so-called *H* centers in alkali halides: a neutral halide atom making the bond with a regular anion, e.g. a Cl_2_^–^ quasi-molecule located at a single anion site was discovered in KCl by the EPR method and related to optical absorption around 3.7 eV^46^). On the other hand, negatively charged anion interstitials, called the *I* centers, are also observed/interpreted in alkali halides with typical absorption bands in UV spectral region (see ^47^). The optical absorption of oxygen dumbbells in metal oxides is reasonably to be expected at higher energies with respect to the *H*-absorption in alkali halides.

Knowledge about single point defects in *cation sublattice* of oxides is clearly insufficient^26,39,48,49^, although cation vacancies are definitely included in more complex structural defects detected by the EPR and other experimental methods^14,19,26,39,40^. Note that at present there is a lack of information on elementary cation defects (single components of an interstitial-vacancy cation Frenkel pair) even in model alkali halide crystals^50^.

To estimate the possible recovery process of structural damage caused by α-Al_2_O_3_ irradiation at the reactor operation temperature, the processes of thermal annealing of the single *F*- and dimer *F*_2_-type defects have been studied both experimentally^25,28–32,37,38^ and theoretically^51–54^. As it was mentioned above, the simulation of the migration of oxygen interstitials, serving as a mobile component in a thermally stimulated recombination of complementary Frenkel defects, has been also analyzed for metal oxides^41,43–45^.

The present paper is devoted to the precise measurements and subsequent simulation in terms of diffusion-controlled reactions of the thermal annealing kinetics of structural anion Frenkel defects created in α-Al_2_O_3_ single crystals by fast fission neutrons. For the first time, along with the annealing of the electronic *F* and *F*^+^ centers (neutral and charged, with respect to the crystal lattice), the annealing kinetics of their complementary mobile defects—oxygen interstitials of two different types (including one recently discovered in a form of O_2_^–^ centers^42^), has been studied and theoretically analyzed. Note that their recombination occurs as the result of diffusion-controlled encounter of mobile interstitials with immobile electron centers which start to migrate at much higher temperatures. Our study is a logical continuation of our recent investigations of radiation damage in magnesium aluminate spinel^55^, magnesium oxide^56^ and corundum^38,42^ started with general aim to achieve a fundamental understanding of different stages of radiation damage kinetics allowing us to predict a long-time radiation tolerance of wide-gap metal oxides promising for various applications in nuclear energetics.

Thus, we have demonstrated for the first time a co-existence of the two types of interstitial oxygen defects–neutral O atoms and negatively charged O^–^ ions, and have shown that they correspond to optical absorption bands peaked at energies of 6.5 eV and 5.6 eV, respectively. Prior to this, in the literature, such a situation had never been considered, although the existence of several charge states of oxygen vacancies has long been beyond doubt and is a well-proven fact.

## Methods

### Experimental

Nominally pure single crystals of α-Al_2_O_3_ (with traces of chromium and iron impurity ions) were grown using the Czochralski method by Union Carbide Corporation. These crystals were irradiated by fast fission neutrons with energy *E* > 0.1 MeV and cumulative fluence *Φ* = 6.9 × 10^18^ n/cm^2^ at neutron irradiation facility of the Oak Ridge National Laboratory. Due to permanent cooling with flowing helium gas, the sample temperature throughout the irradiation did not exceed ~ 330 K.

The spectra of optical absorption were measured at room temperature (RT) by a high-absorbance spectrometer JASCO V-660 in conventional region of 1.5–6.5 eV, while a vacuum monochromator VMR-2 and the hydrogen discharge light source were applied for measurements in near vacuum ultraviolet spectral region up to 8.5 eV. The absorption of a virgin sample was subtracted from the spectrum of a neutron-irradiated crystal and just that difference was regarded as so-called radiation-induced optical absorption (RIOA). The plates about 8 × 8 mm^2^ were cut off parallel to the main *c* crystal axes (in a few cases, perpendicular to *c*) and polished from both sides to optical transparency. In order to stay within experimental limits of optical density (OD) values, OD ≤ 4.0 in different spectral regions, the sample thickness varies from 3 mm to 0.09 mm.

The paramagnetic centers were also investigated using an X-band (9.8 GHz) EPR spectrometer Bruker ELEXYS-II E500. The irradiated samples aligned with different orthogonal crystallographic directions were used while measuring the angular dependences of the EPR spectra needed for origin/structure determination of unidentified defects. A special Bruker program was applied for establishing the defect concentrations in the samples.

Exactly the same regime was applied measuring the temperature dependences of defect concentrations determined via the EPR or optical absorption. The following stepwise annealing procedure was applied: firstly, the irradiated crystal placed into a quartz reactor was preheated to a certain temperature *T*_*pr*_ in an extra pure flowing argon atmosphere; secondly, kept at this fixed *T*_*pr*_ for 10 min; and finally, cooled down to RT by moving the reactor with the sample out of the furnace. Multiple “heating–cooling-measuring” cycles were implemented with the sequential increase of *T*_*p*r_ by 25 to 40 K, while all the RIOA and EPR spectra were measured at RT. The similar thermal annealing procedure was used in our recent studies of radiation damage recovery in radiation-resistant oxides: MgO^56,57^, MgAl_2_O_4_ spinel^58,59^, and Al_2_O_3_ single crystals^38,42^.

### Theoretical

As it is already mentioned, incident particles with above-threshold energy produce pairs of Frenkel defects in oxide materials (including corundum). The observation of the neutral and charged electron centers in oxygen sublattice (the *F* and *F*^+^ centers) using optical and EPR methods also suggests the existence of their complementary *H*^0^ and *H*^−^ interstitials (a superscript describes a charge state of an oxygen interstitial)^42^. However, the main problem in experimental observation of radiation-induced oxygen interstitials is the apparent lack of relevant optical absorption data; the only recent proof of the oxygen dumbbell formation under neutron irradiation of α-Al_2_O_3_ is based on the EPR data^42^.

Recently we have developed a simple model of the bimolecular diffusion-controlled recombination of the *F*-type centers with mobile interstitials in different irradiated ionic solids^51–56,60^, which allows us to extract basic parameters of the interstitial diffusion (the *F*-type centers in corundum are practically immobile below 1500 K^28,30,37^). Note that up to now, the elaborated phenomenological theory considered the involvement of only one type of interstitials in mutual recombination with immobile oxygen-vacancy-containing centers.

**Figure 1:**
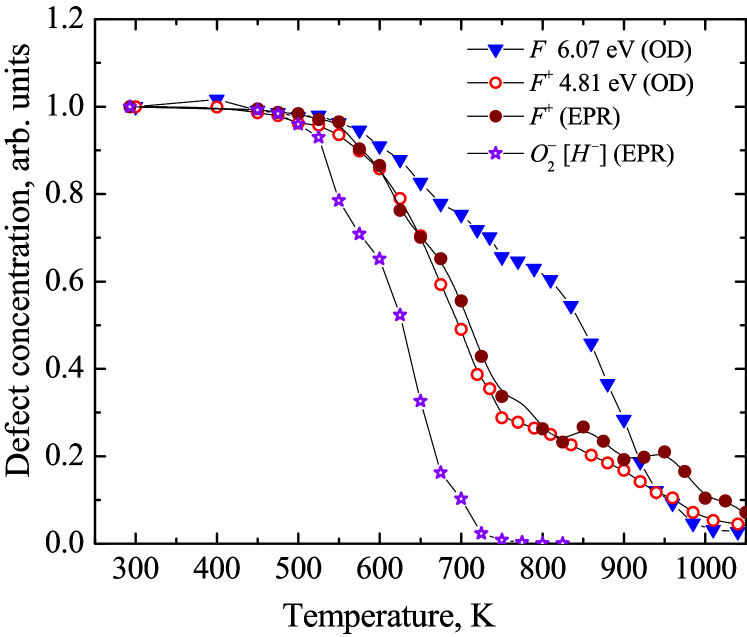
Thermal annealing kinetics of the electron-type *F* and *F*^+^ centers as well as O_2_^−^ dumbbells (that include *H*^−^ interstitials, see for details Sect. 3, text related to Fig. 5). The decay of normalized defect concentrations which were estimated via the absorption band (OD) or EPR signal related to a certain radiation defect (see next Section for details).

However, according to Fig. 1, here we face a more complicated situation with the thermal annealing kinetics of fast-neutron-induced Frenkel defects in corundum. First of all, the kinetics of the charged (only one electron within an oxygen vacancy) and paramagnetic *F*^+^ centers, the concentrations of which were monitored via both the optical absorption and EPR methods (see Sect. 3 for details), are very close that confirms in turn their correctness. At the same time, the concentration of the *F* centers falls down above 600 K essentially slower than that for the *F*^+^ centers, until both types of vacancy-containing defects disappear at about 1000 K. Moreover, both kinetics are not smooth and show a noticeable step around 750 K. On the other hand, the annealing kinetics for the O_2_^−^ dumbbell (the thermal decay of which cause the release of *H*^−^, i.e. a charged oxygen interstitial) demonstrates a rather sharp decay, these radiation defects disappear already around the 750 K step mentioned above for the annealing of the *F* and *F*^+^ centers. All this indicates that our simple model needs a serious revision and very likely, another type of an oxygen interstitial (a neutral one) should be involved as the second mobile recombination component complementary to the *F* centers. In order to find experimental manifestations and identify the oxygen interstitials, here we carefully analyze the measured optical absorption spectra via their decomposition into elementary Gaussian components. Furthermore, we discuss physical meaning of these components, their annealing kinetics will be included into the analysis of the total recombination process.

Since our new model involves *two* types of the electron centers, the *F* and *F*^+^, and two types of interstitials, the *H*^0^ and *H*^−^, there are four possible recombination processes. The situation is simplified due to the fact that the electron centers are immobile until 1500 K and, thus, the diffusion recombination is defined only by the migration parameters of two interstitials, the more so, their migration energies have been theoretically estimated already^43,45^. Another problem is the estimate of the temperature dependent radius for the recombination of the oppositely charged and, thus, attracting each other defects, *F*^+^ and *H*^−^. As we have shown in Supplementary, namely the *F*^+^ − *H*^−^ Coulomb interaction leads to the formation of the gap in the *F* and *F*^+^ decay kinetics.

## Results and discussion

**Figure 2:**
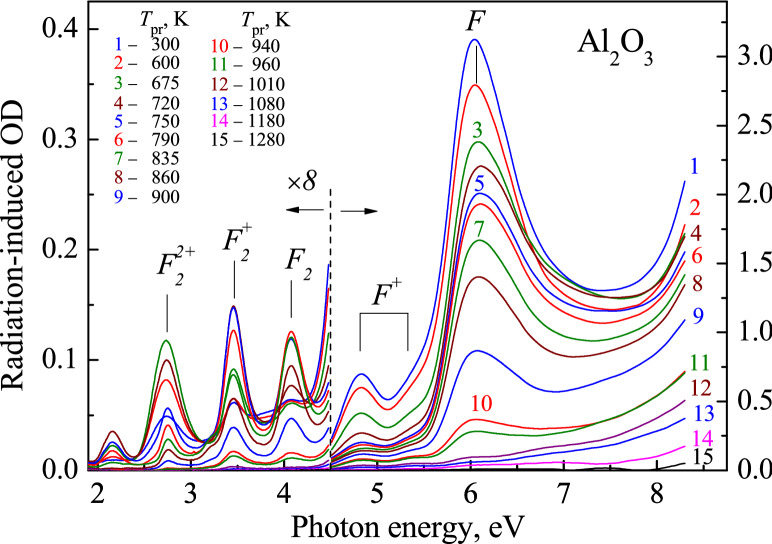
Spectra of RIOA for an Al_2_O_3_ crystal with *d* = 0.09 mm after irradiation with fast fission neutrons (fluence *Φ* = 6.9 × 10^18^ cm^-2^, curve 1) or additional preheating of the same irradiated sample to different temperatures *T*_pr_. All spectra are measured at RT.

**Figure 3:**
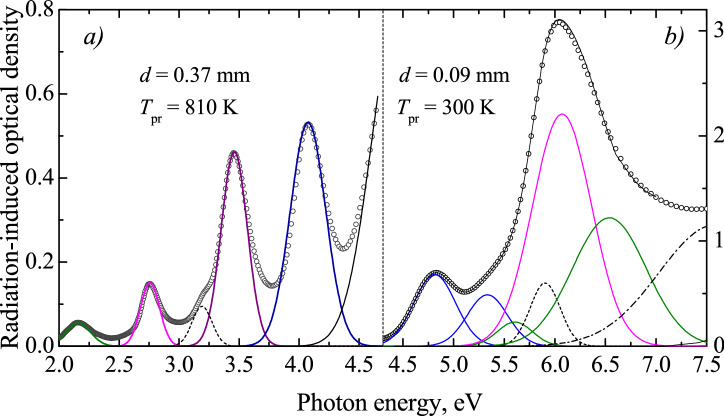
The decomposition of RIOA spectra (curves with symbols, οοο) into Gaussian components (color lines) for the neutron-irradiated α-Al_2_O_3_ single crystals (*Φ* = 6.9 × 10^18^ cm^-2^). Right side—the experimental spectrum is the RIOA measured after irradiation of the sample with *d* = 0.09 mm; left side—the experimental spectrum is the spectrum after preheating of the irradiated sample with *d* = 0.37 mm to 810 K.

**Figure 4:**
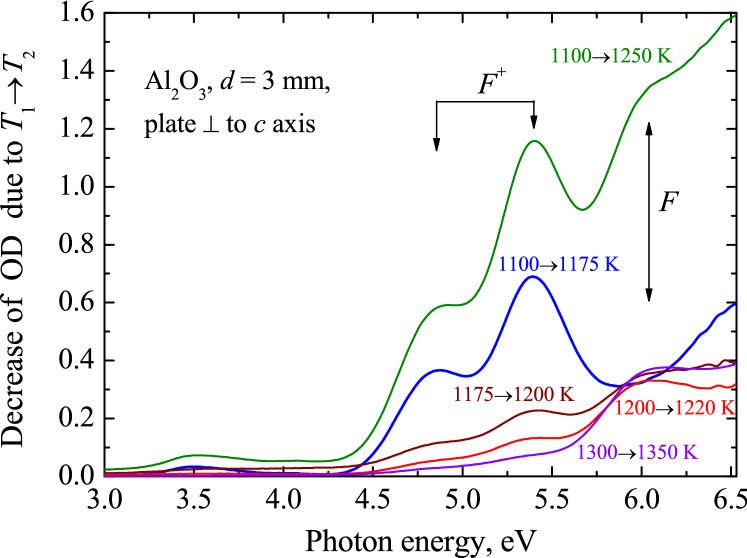
Difference spectra representing the decrease of RIOA due to the preheating of the neutron-irradiated Al_2_O_3_ single crystal (*Φ* = 6.9 × 10^18^ cm^-2^, RT, plate with *d* = 3 mm cut of perpendicular to main *c* axis) from *T*_pr_(1) to *T*_pr_(2).

Figure 2 shows the spectra of RIOA measured in a wide spectral region for a α-Al_2_O_3_ single crystal irradiated by fast neutrons (curve 1) as well as for the same irradiated sample additionally preheated to certain temperatures *T*_pr_. All spectra are measured at the same temperature, RT. Use of the irradiated sample with a thickness of *d* = 0.09 mm allowed us to stay within experimental limit of optical density (OD ≤ 4) in a whole spectral region spread up to the beginning of exciton absorption and band-to band electron transitions (above 8.8 eV and *E*_g_ = 9.4 eV, respectively at helium temperatures^61^). According to the literature data (see ^24,25,28–32,37,38^, the most intense absorption band peaked around 6 eV is related to the neutral *F* centers; absorption bands with the maxima at ~ 5.3 and ~ 4.8 eV are connected with the charged oxygen-vacancy-containing *F*^+^ centers (see Figs. 3 and 4 where both these elementary absorption components are clearly observed), while three weak bands peaked around 2.75, 3.45 and 4.1 eV are ascribed to the simplest anion vacancy aggregates, *F*_2_ dimers in different charge states—*F*_2_^+^ and *F*_2_^2+^ and *F*_2_, respectively.

To estimate separately the contribution of different defects into RIOA, complex spectra could be decomposed into a number of elementary Gaussians. The result of such decomposition is demonstrated in Fig. 3. We tried to minimize the number of Gaussian components of such formal decomposition keeping in mind the data available in the literature. The high-energy part of the spectra corresponds to the 0.09-mm crystal just after irradiation with fast fission neutrons, while the RIOA spectrum after preheating of the irradiated Al_2_O_3_ crystal (*d* = 0.37 mm) to *T*_pr_ = 810 K was used for the decomposition in the region of rather weak absorption of *F*_2_ dimers (2.5–4.5 eV). Note that the use of RIOA spectra after additional preheating to high *T*_pr_ for the decomposition into *F*_2_-type Gaussians allowed to eliminate the influence of “alien absorption” in this spectral region (see for details our recent publication^38^). Positions of the maxima *I*_max_ and FWHM values for the Gaussians of our decomposition and their relevance to a certain defect type (if possible) are listed in Table 1.

**Table 1:** Peak positions and bandwidths of elementary Gaussians and their connection with specific neutron-induced defects (if possible) in α-Al_2_O_3_ crystals.

Gaussian	1	2	3	4	5	6	7	8	9	10	11	12
Peak position, eV	2.159	2.757	3.192	3.46	4.075	4.813	5.333	5.604	5.903	6.071	6.538	7.584
FWHM, eV	0.243	0.189	0.196	0.258	0.349	0.486	0.467	0.409	0.350	0.687	0.859	1.310
Defect designation	*F*_2_^+^^38^	*F*_2_^2+^	*F*_2_^38^	*F*_2_^+^	*F*_2_	*F*^+^	*F*^+^	O_2_^−^ *H*^−^		*F*	H^0^	

The assignment of absorption bands to the *F*-and *F*_2_-type centers is based on literature data^24,25,28–32,37,38^, while oxygen-interstitial-related bands are considered for the first time in the present study (*H*^0^) and for *H*^−^—in our recent publication^42^ (see text for details).

Besides already mentioned absorption bands related to the *F*-type defects (single *F* and *F*^+^ as well as *F*_2_ dimers) there are several other components of decomposition. The component with *I*_max_ = 5.9 eV might be also consistent with the *F*^+^ centers (the possibility of the third absorption band was suggested in the literature). However, our results on the thermal annealing of RIOA have not confirmed the kinetics correlation for the 5.9 eV band and two other UV bands definitely ascribed to the *F*^+^ centers. Note that the *F*^+^ bands with *I*_max_ = 4.82 and *I*_max_ = 5.33 eV are noticeable after decomposition procedure but not in original RIOA spectra (see Fig. 2). However, it is true only if a α-Al_2_O_3_ single crystal was cut off parallel to the main *c* crystal axis (just these neutron-irradiated corundum plates were used for the main optical experiments in the present study). On the other hand, both the *F*^+^-bands (4.8 eV and 5.3 eV) are clearly separated in the difference RIOA spectra (decay of absorption between consecutive preheating to two *T*_pr_) of a neutron-irradiated α-Al_2_O_3_ sample cut off perpendicular to *c* axis (see Fig. 4). The use of a thick sample with *d* = 3 mm allows detecting the RIOA related to the small concentration of the *F* and *F*^+^ centers at high-temperature annealing stage. This result is in full agreement with the earlier study of polarized optical absorption of neutron-irradiated Al_2_O_3_ crystals^25,28^. According to Ref. 33, the Gaussians peaked at ~ 3.2 eV and ~ 2.15 eV could be tentatively considered as the second absorption bands related to neutral and single-charged *F*_2_ dimers, respectively.

**Figure 5:**
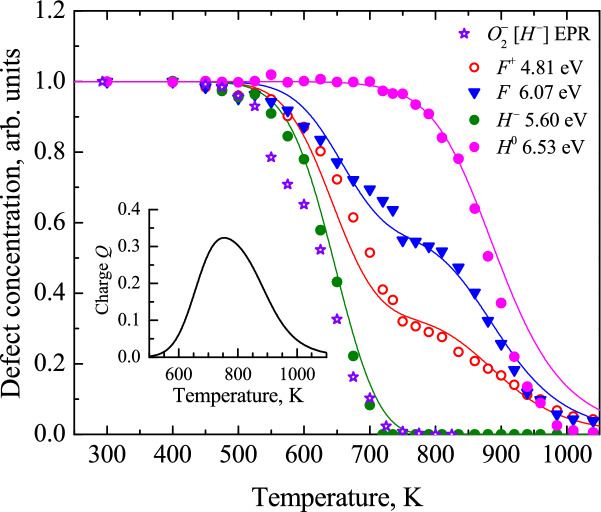
The normalized experimental annealing kinetics for four types of primary defects (stars for EPR, other symbols for optics)) and their theoretical analysis (full lines). Insert shows the electronic charge released due to recombination of *F*^+^–*H*^0^ and *F*–*H*^−^ pairs. The values of OD at *I*_max_ were taken to follow the annealing of the corresponding defects via optical absorption. *H*^−^ arise via the thermal decay of paramagnetic O_2_^−^ defects.

The Gaussian band with *I*_max_ = 5.6 eV is ascribed to the *H*^−^ oxygen interstitials, on the basis of our recent results^42^. For the first time in metal oxides, the EPR signal of oxygen interstitials in a form of a superoxide ion O_2_^−^ located in a regular lattice region, i.e. without interaction with any other structural/impurity defect, was revealed in a neutron-irradiated α-Al_2_O_3_ crystal. The pulse annealing of the EPR signal of such dumbbell defects was measured as well; this decay kinetics clearly correlates with the thermal annealing of RIOA Gaussian component peaked at 5.6 eV (see also Fig. 5). Considering an obvious coincidence of the *F*^+^ center annealing curves measured via the EPR signal or the corresponding absorption band (see also Fig. 1), the Gaussian with *I*_max_ = 5.6 eV was attributed in^42^ to the charged *H*^−^ interstitial (a part of O_2_^−^ ), a complementary defect to vacancy-containing *F*^+^ center. The interpretation of the band at 6.5 eV is discussed below.

Precise experimental study of thermal annealing/transformation of different radiation defects can be performed with the use of the decomposition of RIOA into elementary Gaussian components, each of which serves as a measure of a certain defect type. Such decomposition procedure was applied to the RIOA spectra measured at RT after preheating to each *T*_pr_. As a result, the temperature dependence of the concentration of different structural defects could be constructed and analysed. Note that our recent studies of the annealing of radiation-induced damage in MgAl_2_O_4_^55^, MgO^56^ and Al_2_O_3_^38,42^ confirm that the annealing curves practically coincide in shape if the concentration of the corresponding radiation defects after preheating to certain temperatures is taken proportional to the integrated area *S* or the Gaussian peak intensity (OD at *I*_max_). The real concentration of the radiation defects in the samples could be roughly estimated using the Smakula-Dexter formula (uncertainty is connected with the unknown oscillator strength value). In case of paramagnetic nature of the defect, a special Bruker program was applied for establishing the defect concentration—in our samples the concentration of the EPR-active *F*^+^ defects equals 1 × 10^18^ cm^-3^ and practically coincides with that for the *H*^−^ oxygen interstitials (being a part of O_2_^−^)^42^. In general, a combined use of optical absorption and EPR methods for the identification of structural defects and study of their thermal annealing is very fruitful.

We have applied such experimental approach to the study of the thermal annealing of radiation damage in α-Al_2_O_3_ single crystals exposed to fast fission neutrons (*Φ* = 6.9 × 10^18^ cm^-2^). The experimental annealing curves, describing the preheating temperature dependence of the concentration of different defects associated with typical elementary absorption bands (see Gaussians in Fig. 3 and Table 1), have been constructed. In the present paper, we focused on the theoretical analysis of the annealing kinetics of Frenkel pairs of anion defects. Similar to alkali halides, the multistage annealing curves of anion-vacancy-containing Frenkel defects (see^47,62^) could be explained by the presence of Frenkel pairs of both neutral and charged anion defects involved in recombination process (so-called *α-I* and *F–H* pairs in alkali halides).

Figure 5 shows the experimental annealing curves (symbols) constructed using OD values at *I*_max_ for four elementary Gaussian components of RIOA listed in Table 1. The pulse annealing of the EPR signal of the O_2_^−^ oxygen dumbbell interstitials (stars) is demonstrated as well. It was mentioned already in Sect. 2.2 that the annealing of the *F* and *F*^+^ centers occur in two main stages: from approximately 500 K up to 750 K and then at 750 − 1100 K. Note that the annealing curves for both absorption bands ascribed to the *F*^+^ centers coincide with high accuracy (the band with *I*_max_ = 4.81 eV is used in Fig. 5). The decay of the *F*^+^ EPR signal clearly repeats two-stage annealing for the *F*^+^ absorption band (see Fig. 1 and Ref ^37^.). The similar coincidence is true for the annealing curves of the O_2_^−^ EPR signal and the absorption Gaussian with *I*_max_ = 5.6 eV that, in turn, confirms attribution of both signals (EPR and absorption) to the same radiation defect—the charged oxygen interstitial, *H*^−^^42^. It is worth noting that the EPR signals of both detected paramagnetic centers always measured at RT remain permanent throughout the whole multistage annealing process (see^42^ for details).

Full lines in Fig. 5 present the results of our theoretical fitting for a novel model with two becoming mobile oxygen interstitials, the *H*^0^ and *H*^−^, and four types of defect interaction/kinetics (see more details and parameters used in Supplementary Information). Note that we used theoretical estimates for the migration energies of the neutral and charged interstitials, 1.2 eV and 0.8 eV, respectively, close to those suggested in Refs ^43,45^.). To provide theoretical analysis of such four interrelated kinetics and to fit correctly the experimental multistage annealing curves for the *F* and *F*^+^ centers, we assumed that the Gaussian peaked at ~ 6.5 eV is associated with the neutral *H*^0^ oxygen interstitials (the Gaussian with *I*_max_ = 5.6 eV is already attributed to the O_2_^−^ defects, associated with the *H*^−^ interstitials).

Figure 5 demonstrates an excellent agreement between experiment and theory; all four annealing kinetics peculiarities are reproduced here. The charged interstitials *H*^−^ (released after decay of the O_2_^−^ centers) begin to migrate first and rapidly disappear at ~ 750 K, recombining initially with the *F*^+^ (complementary defects) and then with the *F* centers. The delay (~ 50–100 K) between the *H*^−^ and *F*-type center decay can be explained as follows. The EPR-active superoxide O_2_^−^ ion (just the annealing of its EPR signal is shown in Figs. 5 and 1) revealed for the first time in Ref.^42^ is a hole-type molecular center (oxygen dumbbell) that incorporates one regular and one interstitial oxygen. Its decomposition (the first step) results in the release of an oxygen interstitial (in our opinion, in a form of *H*^−^), which migrates towards immobile electron centers and recombines with one of them. This second reaction step requires additional time and it is seen in Fig. 5 as a shift in the temperature.

The kinetics of the electron *F* and *F*^+^ center annealing also show the gap due to the above-mentioned Coulomb attraction in the *F*^+^–*H*^−^ pairs which strongly accelerates their mutual recombination. On the other hand, the *F* and *F*^+^ kinetics approach each other around 900 K due to relative weakening of the Coulomb interaction kinetics (see more details in Supplementary). Unlike charged interstitials, the neutral ones, *H*^0^ are much more stable and start to migrate only around 750–800 K.

Thus, the step observed around 750 K in both the *F* and *F*^+^ kinetics arises due to *interplay* between the *H*^*−*^ and *H*^0^ interstitials; all *H*^*−*^ interstitials already undergo fast recombination with immobile counterparts till this temperature point (total disappearance of the O_2_^−^ EPR signal), whereas neutral *H*^0^ oxygen interstitials only start their slow migration. Lastly, intensive recombination of the mobile *H*^0^ with the electron *F*^+^ and *F* centers begins around 800 K. Unfortunately, the excellent fit of experimental data with the theoretical simulation of the annealing processes is up to now the only (but rather serious) argument for the 6.5 eV Gaussian attribution to the *H*^0^ oxygen interstitials. Experimental proof of this suggestion as well as additional confirmation of the belonging of the RIOA component with *I*_max_ = 5.6 eV to the charged *H*^*−*^ interstitials still lies ahead.

The above-described scenario allows us also to analyze the charge balance in the system. Initially, the concentrations of positively and negatively charged Frenkel defects in neutron-irradiated α-Al_2_O_3_ single crystals are assumed to be equal (system electroneutrality). However, in the process of the *F*^+^–*H*^0^ and *F*–*H*^−^ pair recombination free electrons and holes are released, respectively, and trapped somewhere in the crystal. For instance, excess charge carriers could cause recharging of impurities (some rise of Cr^3+^ impurity ion concentration is detected via the EPR method in our samples at temperatures above 450 K, when the decay of hole-containing O_2_^−^ starts) or aggregate defects, including *F*_2_ dimers (according to Ref. 32, additional electrons should be involved in the sequent transformation F_2_^2+^  → F_2_^+^  → F_2_ starting above 650 K). The insert in Fig. 5 demonstrates that the resulting charge corresponds to the excess of electrons constituting ca. one third of the initial charge of the interstitials.

## Conclusions

The recovery of radiation damage under thermal annealing of α-Al_2_O_3_ single crystals irradiated with fast fission neutrons (*Φ* = 6.9 × 10^18^ *n*/cm^2^) has been thoroughly studied by means of optical absorption and EPR methods. The precisely measured annealing kinetics for different anion Frenkel defects were compared with those simulated in terms of diffusion-controlled bimolecular reactions. For the first time, the elaborated phenomenological theory considers the involvement of *two types of mobile oxygen interstitials* (charged *H*^−^ and *H*^0^) in their mutual recombination with complementary immobile oxygen-vacancy-containing centers (the *F* and *F*^+^). The concentrations of the neutral and charged Frenkel pairs are comparable in our samples. Our four defect interrelated kinetics model allows us to reach an excellent agreement between experiment and theory and to explain the two-step behavior of the annealing of the *F* and *F*^+^ centers. Our model gives the unique set of parameters (see Supplement Information for details) able to reproduce simultaneously all four annealing kinetics shown in Fig. 5. Based on the coincidence of experimental and theoretical annealing kinetics, we tentatively attribute the elementary absorption band peaked at 6.5 eV to the *H*^0^ oxygen interstitials and confirm our recent assignment of the Gaussian with *I*_max_ = 5.6 eV to the charged *H*^−^ interstitials. At the same time, the additional experimental proof of the linkage of these radiation-induced absorption bands to oxygen interstitials in different charge states still lies ahead. The suggested analysis of the diffusion-controlled kinetics of radiation defect transformation and annealing could be applied to a wide class of oxide and nitride materials^8–10,13,36,63–71^.
